# Regularization for Unsupervised Learning of Optical Flow

**DOI:** 10.3390/s23084080

**Published:** 2023-04-18

**Authors:** Libo Long, Jochen Lang

**Affiliations:** Faculty of Engineering, University of Ottawa, Ottawa, ON K1N 6N5, Canada

**Keywords:** self-supervised training, teacher–student learning, regularization, optical flow, scene flow

## Abstract

Regularization is an important technique for training deep neural networks. In this paper, we propose a novel shared-weight teacher–student strategy and a content-aware regularization (CAR) module. Based on a tiny, learnable, content-aware mask, CAR is randomly applied to some channels in the convolutional layers during training to be able to guide predictions in a shared-weight teacher–student strategy. CAR prevents motion estimation methods in unsupervised learning from co-adaptation. Extensive experiments on optical flow and scene flow estimation show that our method significantly improves on the performance of the original networks and surpasses other popular regularization methods. The method also surpasses all variants with similar architectures and the supervised PWC-Net on MPI-Sintel and on KITTI. Our method shows strong cross-dataset generalization, i.e., our method solely trained on MPI-Sintel outperforms a similarly trained supervised PWC-Net by 27.9% and 32.9% on KITTI, respectively. Our method uses fewer parameters and less computation, and has faster inference times than the original PWC-Net.

## 1. Introduction

Estimating optical flow, which is the apparent motion of objects in a scene, is a basic task in computer vision that has many applications [1,2,3,4,5,6,7]. In recent years, convolutional neural networks (CNNs) [8,9,10] have been used to learn optical flow from data, and they have shown better performance and efficiency than traditional methods based on variational models [11,12,13,14]. However, learning optical flow from data poses some challenges, depending on the type of data and the learning paradigm. In this paper, we focus on the unsupervised learning of optical flow from real images without ground-truth annotations. We review the existing methods and challenges in this area, and propose a novel approach to improve the accuracy and robustness of optical flow estimation.

To cope with the difficulties of the unsupervised learning of optical flow, previous methods have proposed various techniques to reduce the errors caused by occlusion, illumination changes, textureless regions, and motion boundaries [15,16,17,18,19,20,21]. However, these techniques are often specific to certain types of errors and may introduce noise or complexity to the network. In this paper, we propose a different approach that does not rely on these error-specific techniques, but instead uses a general regularization strategy in a novel teacher–student framework.

The teacher–student framework is a common strategy for unsupervised learning, where a teacher network provides guidance to a student network based on some learned representation. However, a limitation of this strategy is that the teacher and student networks do not improve together during the training process. If the student network learns a better representation than the teacher network, the guidance from the teacher network becomes less effective. Some methods have tried to address this issue by using a co-teaching strategy [22,23], where two networks teach each other iteratively, but this comes at the cost of training two separate networks. In this paper, we introduce a novel shared-weight teacher–student strategy, where the teacher and student networks share the same network weights but differ in the use of an additional regularization module. The network with regularization acts as the teacher, and the network without regularization acts as the student. In this way, both networks are updated simultaneously via backpropagation and they benefit from each other’s improvements.

Another aspect that we investigate in this paper is the generalization ability of optical flow networks, which is often limited in unsupervised learning methods. We hypothesize that regularization methods can enhance the generalization ability of unsupervised optical flow networks, as they do for image classification and semantic segmentation networks [24,25]. We test different regularization methods in our teacher–student learning strategy and show that they have some positive effects on the performance. However, we also discover that our novel content-aware regularization (CAR) module improves the generalization ability more than existing methods.

The CAR module is a simple but effective way to enhance and diminish the features of the network randomly during training in the teacher model, and then remove it during testing. The CAR module works as follows: given an input feature x, it splits x into k subsets, randomly selects one subset, and computes residuals only from that subset. The CAR module is content-aware because it learns a convolutional layer based on the features without regularization. We argue that this idea can prevent the co-adaptation of channels and help the network learn more discriminative features in each channel. As a result, the shared-weight network improves for both the teacher and student models.

The proposed CAR module has several advantages: it has a low memory footprint, it supports real-time computation and end-to-end training, and it can be easily integrated into different tasks and networks. We implement CAR in a modified version of PWC-Net [26], called PWC-Lite [27], which is a small unsupervised optical flow network. We also extend our method to scene flow estimation, which is a more challenging task that involves the estimation of both optical flow and depth. The experimental results show that our method significantly improves on the original PWC-Net model without any additional space and time costs during inference. Our method solely trained on MPI-Sintel outperforms supervised PWC-Net by 27.9% and 32.9% on KITTI, respectively. To summarize, our main contributions are as follows:

- We propose a novel and effective teacher–student unsupervised learning strategy for optical flow and scene flow estimation, where the teacher and student networks share the same weights but differ in the use of a content-aware regularization module.
- We experimentally show that a PWC-Net model trained with our unsupervised framework outperforms all other unsupervised PWC-Net variants on standard benchmarks. The multi-frame version surpasses supervised PWC-Net with lower computational costs and using a smaller model.
- A PWC-Net model trained with our method shows superior cross-dataset generalization compared to supervised PWC-Net and unsupervised ARFlow.

## 2. Related Work

### 2.1. Supervised Optical Flow Methods

Supervised methods learn optical flow based on ground-truth. FlowNet [28] is the first end-to-end convolutional optical flow network. Large displacements are estimated in a coarse-to-fine process by SpyNet [9]. PWC-Net [26] and LiteFlowNet [10] proposed a feature pyramid, warping, and cost volume architecture with many follow-ups [29,30,31,32,33,34]. RAFT [35] improved the estimation of flow using a 4D pixel-to-pixel correlation volume and an iterative refinement network, achieving state-of-the-art performance. However, these methods heavily rely on large scale synthetic datasets such as FlyingThings3D and expensive ground-truth labels. In this paper, we focus on unsupervised learning, which does not require ground-truth labels.

### 2.2. Unsupervised Optical Flow Methods

Learning optical flow without ground-truth labels makes basic assumptions regarding brightness constancy and spatial smoothness [36]. In unsupervised learning, the photometric loss calculates the difference between the first image and the warped second image based on the predicted flow. Different strategies were proposed to improve accuracy, e.g., via occlusion handling [15,16], a multi-frame formulation [37], data distillation [38,39,40], the use of the epipolar constraint [41], depth constraints [19,20,21,42], and data augmentation [27]. UFlow [43] evaluates and integrates multiple constraints into a framework. In UPFlow [18] a pyramid distillation loss is proposed for unsupervised learning achieving state-of-the-art performance. MDFlow [44] uses PWC-Net and RAFT in a student–teacher configuration. Marsal et al. [45] propose a brightness correction network that calculates a correction map based on the reference image, the flow-warped second image, the current flow estimate, and an occlusion map, demonstrating an improvement in the unsupervised training of various networks including RAFT. SMURF [46] has used RAFT as a backbone network and guides the network using full-resolution multi-frame self-supervision, which offers impressive accuracy. However, this framework is extremely expensive. For each dataset, SMURF pre-training takes 1 day to converge on 8 GPUs. Then, Stone et al. generate labels by training separate tiny models for all images (i.e., ≈2000 models for MPI-Sintel). The tiny models are trained with full-resolution images, which takes an extremely long time if trained on a single machine. Finally, the network is fine-tuned with a combination of an unsupervised and a self-supervised loss. Because an optical flow network acts typically as a fundamental building block in high-level applications, our goal is to support low memory, real-time computing and end-to-end training. We conduct experiments on a small PWC-Net variant, PWC-Lite [27], and we do not require an elaborate and resource-intensive training strategy.

### 2.3. Regularization in CNNs

To increase the generalization and to prevent the CNN layers from co-adapting, many regularization techniques have been proposed, such as data augmentation [47] and dropout [48,49].

Dropout is a popular regularization technique in deep neural networks. Many researchers have proposed variants of dropout for CNNs. SpatialDropout [50] uses dropout across entire features maps. DropBlock [24] removes part of the semantic information by randomly dropping a square region of the feature map. StochasticDepth [51] randomly drops a subset of layers during training while employing the full network when testing. Recently, DropCluster [52] finds clusters of correlated features in outputs of convolutional layers, then it randomly drops the clusters during training. However, in experimental evaluations, those methods are shown to be effective for the classification problem. In our observation, in optical flow, and indeed, in most pixel matching tasks, dropout seems to be avoided; at least, we are not aware of its successful application.

Data augmentation is widely used in CNN methods [53,54] to improve training. FlowNet2 [55] proposed the first systematic data augmentation scheme for an end-to-end optical flow network. ScopeFlow [56] put forward effective data augmentation by dynamically increasing the cropping rate during training. Recently, ARFlow [27] has used the prediction of flow for the original images to guide the flow for augmented images by transforming the original prediction.

### 2.4. Teaching Strategy

The unsupervised learning of optical flow has been an active research area in recent years. Many methods have adopted the teacher–student strategy to improve the performance and generalization of unsupervised networks. SelFlow [39] proposed a self-supervised learning framework that alternates between a teacher model and a student model. The teacher model is trained with a self-consistency loss that measures the consistency between the forward and backward flows. The student model is trained with a self-distillation loss that minimizes the difference between the teacher and student predictions. ARFlow [27] introduced an adaptive robust loss function that adapts to different regions of the image based on the teacher’s confidence. The teacher model is trained with a photometric loss and a smoothness loss, while the student model is trained with an adaptive robust loss and a distillation loss. MDFlow [44] used multiple teacher models with different levels of difficulty to guide the student model. The difficulty level is determined by the image quality and the motion magnitude. The student model is trained with a weighted combination of losses from different teachers. Self-Guidance [57] improved the generalization of deep neural networks via knowledge distillation. The teacher model is trained with a self-guidance loss that encourages the network to learn from its own predictions on unlabeled data. The student model is trained with a distillation loss that transfers the knowledge from the teacher to the student. Our method differs from these methods in several aspects. First, we use a shared-weight network for both the teacher and student models, which reduces the memory and computational costs and enables the simultaneous improvement of both models. Second, we propose a content-aware regularization module that randomly enhances and diminishes partial features during training in the teacher model. This module helps to break the co-adaptation between channels and to learn more discriminative features for optical flow estimation. Third, we demonstrate that our method can be easily extended to scene flow estimation by adding a depth estimation branch and applying our regularization module to both branches.

## 3. Methods

### 3.1. Network Structure

Given a pair of RGB images $I_{t}$,$I_{t+1}$, the estimation of optical flow can be formulated as:

(1) $$f\left(p\right)=A\left(I_{t},I_{t+1},\theta \right),$$

where f(p) is the resulting flow per pixel *p*, *A* is the estimation network, and θ are the learnable parameters of the network.

**Figure 1:**
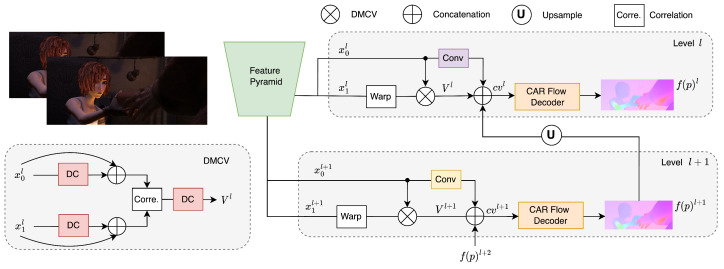
Overview of our unsupervised optical flow method with content-aware regularization (CAR). We present two levels of the pipeline (DMCV is deformable modulation cost volume; see Section 3.1).

Flow estimation networks commonly use an encoder–decoder structure. We use a PWC-Net variant (unsupervised) as the backbone [27,39]. The optical flow is estimated in a coarse-to-fine manner. The network contains a shared-weight Siamese feature pyramid which extracts feature maps at different scales from images. At level *l*, the feature map of the second image $I_{t+1}$ is warped by the predicted flow $f{\left(p\right)}^{l+1}$ of the (previous) coarser level. Then, the cost volume $V^{l}$ is calculated using correlation, which represents the matching costs of pixels between images. The decoder module with shared weights at all levels takes the feature map of $I_{t}$, the upsampled flow $f{\left(p\right)}_{↑}^{l+1}$, and the cost volume $V^{l}$ as inputs and predicts the optical flow $f{\left(p\right)}^{l}$ of level *l*. Finally, the upsampled optical flow $f{\left(p\right)}_{↑}^{l}$ is input to the next finer level. Figure 1 shows the architecture of our method. We use PWC-Lite [27,39] as a backbone, with some modifications.

Previous works [58,59,60] use deformable convolution to deal with the problem of small objects disappearing in low-resolution feature maps. Inspired by that, we use a deformable modulation cost volume (DMCV) to recover the distortion and artifacts of small objects using a dense connectivity motion extractor based on deformable convolution (see Figure 1):

(2) $$y_{t}^{l}=Concat\left(\left[x,DC\left(x\right)\right]\right)$$

(3) $$V^{l}=DC\left(C\left(y_{t}^{l},W\left(y_{t+1}^{l}\right)\right)\right),$$

where *x* and *y* indicate the feature and dense feature map, DC is the deformable convolutional layer, and *C* is the correlation operation. The output flow can be formulated as:

(4) $$cv^{l}=Concat\left(\left[conv\left(x_{t}^{l}\right),f{\left(p\right)}_{↑}^{l+1},V^{l}\right]\right),$$

(5) $$f{\left(p\right)}^{l}=E\left(cv^{l}\right),$$

where *E* presents the optical flow decoder module and conv is a convolution layer.

**Figure 2:**
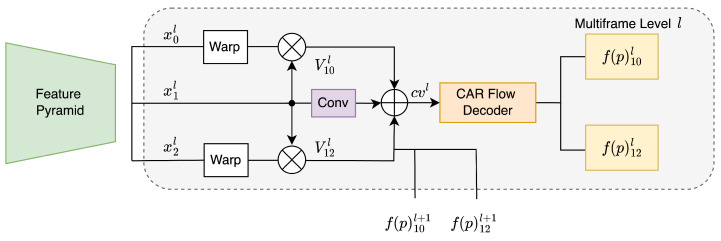
Level *l* of our multi-frame network (see Figure 1 for definitions).

We optionally extend our method into a three frames network by adding the extra backwards flow from the next frame and its corresponding cost volume as sketched in Figure 2. We follow the same multi-frame architecture as ARflow [27], except we added our DMCV and CAR module.

### 3.2. Content-Aware Regularization Module

**Figure 3:**
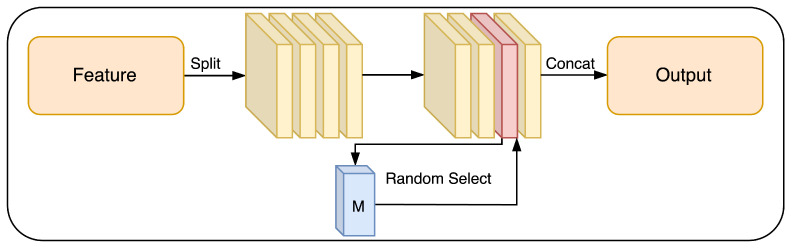
CAR block. The feature map is split into the *k* subset. We randomly choose one subset (red in the graph) to enhance using a residual convolutional module.

The main idea of our method is to use only one network, but to use it twice, once as the teacher network with the CAR module and once without the CAR module as the student network in our shared-weight teacher–student strategy. Only the teacher network uses our CAR module, which we introduce next. Given a feature map, $e\in R^{N\times D}$, where N=W×H. *W* and *H* refer to the width and height of the feature map, respectively, and *D* indicates the depth of the feature map. We first split *e* into *k* subsets $v^{1},\ldots ,v^{k}$ by its depth dimension, where $v^{i}\in R^{N\times D/k}$ is the *i*-th subset of the feature map. Then, we select a subset *j* randomly and compute the new feature map via:

(6) $$\begin{array}{c} \begin{array}{c} v^{1},\ldots ,v^{k}=Split\left(e\right) \end{array} \end{array}$$

(7) $$\begin{array}{c} \begin{array}{c} j=Random(1,k), \end{array} \end{array}$$

(8) $$\begin{array}{c} \begin{array}{c} M=Conv\left(v^{j}\right) \end{array} \end{array}$$

(9) $$\begin{array}{c} \begin{array}{c} {\tilde{v}}^{j}=v^{j}\left(1+M\right), \end{array} \end{array}$$

(10) $$\begin{array}{c} \begin{array}{c} \tilde{e}=concat\left(\left[v^{1},\ldots ,{\tilde{v}}^{j},\ldots ,v^{k}\right]\right) \end{array} \end{array}$$

where *M* is a learnable content-mask which is applied to the *j*-th subset of the feature map *x*. Since *M* is randomly applied to only a subset of *e*, it stops the channels from co-adapting. Random(1,k) in Equation (7) produces a uniform random number in the range [1, *k*] (see Figure 3).

### 3.3. Shared-Weight Teacher–Student Strategy

**Figure 4:**
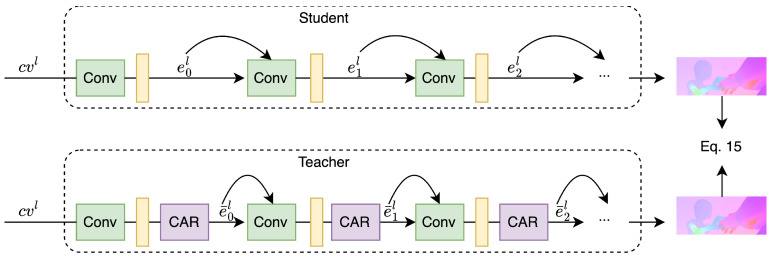
Flow decoder during prediction (**top**) and with CAR during training (**bottom**).

During training, we feed each image pair $I_{t},I_{t+1}$ through the network twice, once for teaching by invoking the CAR module, and once without, for the student. The decoder for the student is shown in the upper part of Figure 4. Given an input $cv^{l}$ as defined in Equation (4), the optical flow decoder module generates features $e_{i}$ (*i* is the index of the hidden layer) using densely connected convolutions in each decoder layer *i*, which can be formulated (dropping the layer superscript *l* for clarity) as

(11) $$e_{i}^{\prime }=CR\left(e_{i-1}\right)$$

(12) $$e_{i}=Concat\left(e_{i}^{\prime },e_{i-1}\right),$$

where *CR* is a convolutional layer, followed by a LeakyReLu activation function. This decoder structure is shown in the upper part of Figure 4. The lower part of Figure 4 shows the structure of the decoder for teaching.

(13) $$e_{i}=Concat\left(CAR\left(e_{i}^{\prime }\right),e_{i-1}\right)$$

where function CAR() indicates Equations (6)–(10). The architecture allows us to choose the number of affected channels by selecting *k*.

The CAR module is randomly applied to a subset of features, breaking the co-adaption between channels. In this strategy, the networks for student and teacher are the same except for the CAR module as they share weights, and the common network is trained together instead of sequentially. During the training, if the student learns a better representation guided by the teacher, the teacher can also benefit because of shared-weight parameters.

**Figure 5:**
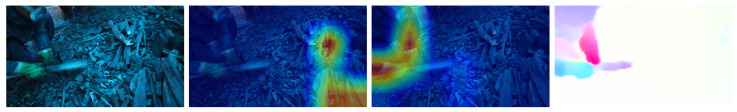
Visualization of CAR. The second column shows regions that are emphasized by the projection in CAR (large positive values of *M*, see Equation (9)). The third column shows regions that are diminished (large negative values of *M*), respectively. The fourth column shows the estimated flow via our method. The result is from a generalization study on Vimeo-90K [61].

We conduct an informal study on the generalization abilities of a PWC-Lite model trained with CAR on Vimeo-90K [61]. To show how CAR improves on the performance, we use a heatmap to visualize what regions CAR enhances and diminishes (see Figure 5). The first column shows an overlay of the input images. The input images only contain one moving object, which is the worker with a static background. The second column in Figure 5 shows regions that are emphasized by the projection in CAR, and the third column shows regions that are diminished.The fourth column shows the estimated flow. We can see that the projection in CAR emphasizes some dark background areas. On the other hand, CAR diminishes some areas with large motions, preventing co-adaption for large displacement. Co-adaption could otherwise potentially lead to incorrect results for regions with small motions.

### 3.4. Regularization and Unsupervised Loss

#### 3.4.1. Content-Aware Regularization

During training, the shared-weight teacher–student strategy forwards data through the network twice, with and without invoking the CAR module. Then, we obtain two different predicted flows, $f^{r}\left(p\right)$ (with CAR module) and $f^{o}\left(p\right)$ (without CAR module). As shown in Figure 4, the flow decoders with and without the CAR module share the same layers, except for the CAR module, which is turned on and off. Therefore, $f^{r}$ is different from $f^{o}$. We regularize the predicted flow by minimizing the difference of two forward passes, i.e.,

(14) $${𝓛}_{ca}=\sum_{p}\psi \left(f^{r}\left(p\right)-f^{o}\left(p\right)\right),$$

where $\psi ={\left(|x|+\epsilon \right)}^{q}$ is a robust function proposed in DDflow [38] with ϵ=0.01 and q=1.

#### 3.4.2. Level Dropout as Regularization

In general, at each level *l*, our method predicts the flow $f^{l}$ based on the coarser-level flow $f^{l+1}$, but we also propose to use level dropout as regularization. We use the finest flow as pseudo labels and supervise a level dropped flow. A similar idea can be found in Uflow [43] but with the main difference being that we propose to also use a corresponding extra regularization loss. Specifically, we predict the flow with the same data twice: First, we randomly drop the calculation of an intermediate flow at pyramid level *i*, and instead pass the resized flow $f^{i+1}$ to the level i−1. The resulting flow calculated with a dropped level is written as $f^{drop}$ in the following. Second, we predict the flow $f^{o}$ by going through all the levels of the pyramid from Levels 6 to 2. Let S() be the stop-gradient, and then the loss function ${𝓛}_{ld}$ can be written as

(15) $${𝓛}_{ld}=\sum_{p}\psi \left(S\left(f^{o}\left(p\right)\right)-f^{drop}\left(p\right)\right).$$

In practice, we also use $f^{r}$, i.e., the flow calculated with the CAR module turned on, to guide $f^{drop}$ as a challenging case with a small probability, which is

(16) $${𝓛}_{rd}=\sum_{p}\psi \left(S\left(f^{r}\left(p\right)\right)-f^{drop}\left(p\right)\right).$$

**Figure 6:**
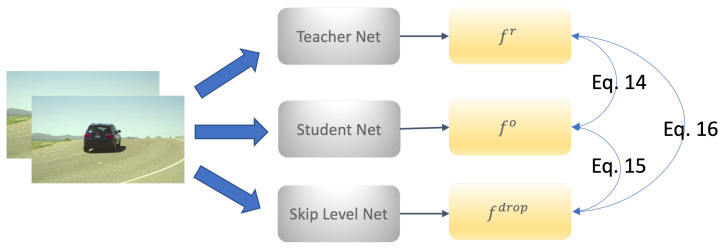
Flow chart illustrating the teacher–student configurations used in our method (see Section 3.4 for details).

The overall regularization loss is a combination of the above three loss functions in Equations (14)–(16). Thus, our shared-weight teacher–student framework will have multiple forward passes in one training step (see Figure 6). The overall loss is

(17) $${𝓛}_{cr}=\alpha \left({𝓛}_{ca},\mu_{1},\delta_{1}\right)+\alpha \left({𝓛}_{ld},\mu_{2},\delta_{2}\right)+\alpha \left({𝓛}_{rd},\mu_{3},\delta_{3}\right),$$

where α() randomly uses the loss in a training step with a fixed probability $\mu_{i}$. In each training step, we generate a uniform random number $\delta_{i}$ in [0,1].

(18) $$\alpha \left({𝓛}_{i},\mu_{i},\delta_{i}\right)=\left\{\begin{array}{cc} {𝓛}_{i}, & \mathrm{if}\;\delta_{i}\leq \mu_{i}\\ 0, & Otherwise \end{array}\right\}$$

#### 3.4.3. Overall Unsupervised Loss

The overall unsupervised loss is then formulated as

(19) $${𝓛}_{all}={𝓛}_{ph}+w_{sm}*{𝓛}_{sm}+w_{au}*{𝓛}_{au}+w_{cr}*{𝓛}_{cr}$$

where ${𝓛}_{ph}$ is the photometric loss, ${𝓛}_{sm}$ is the smoothness regularization loss [62], and ${𝓛}_{au}$ is the augmentation regularization loss [27].(We use the same augmentation setting as ARFlow.) In practice, we set $w_{sm}$ = 1, $w_{au}$ = 0.01, and $w_{cr}$ = 0.01.

## 4. Experiments

### 4.1. Implementation Details and the Use of Datasets

We conduct experiments on two commonly used optical flow benchmarks: MPI-Sintel [63] and KITTI (including KITTI 2012 [64] and KITTI 2015 [3]). We follow the same augmentation setting as in previous methods [27,38,39].

Our results on the MPI-Sintel benchmark protocol are obtained via pretraining on KITTI and then fine-tuning on Sintel. We do not pretrain on Sintel raw. Sintel raw contains the raw scenes for the benchmark, and hence, pretraining on Sintel raw may cause data leakage into the test set. For the KITTI benchmark, we pretrain our network with the KITTI raw dataset (we discard all scenes that contain images that appear in the optical flow KITTI benchmarks) and finetune the network on the KITTI multi-view training dataset. This is the same dataset configuration for KITTI, as used by previous works [27,38,39]. We also implement our method in the related dense pixel matching task of unsupervised scene flow estimation.

We implement our method with PyTorch [65]. For all training, we use the Adam optimizer [66] with $\beta_{1}=0.9,\beta_{2}=0.99$. We first train our method for 1000 k: please check if k is unit, if so, please add space before. iterations with a learning rate of 0.001 and a batch size of 4, followed by a 400 k iteration with a learning rate of 0.005 and a batch size of 1. We increase the crop resolution in the second stage for KITTI from (256, 832) in the first 1000 k to (320, 1216) in the following 400 k. The total numbers of our model parameters are 2.78 M (for two-frame) and 2.97 M (for multi-frame) during training. Since the CAR module (0.12 M) is dropped after training, the final model in predicting the optical flow for a pair of images with a resolution of (448, 1024) is even smaller than during training.

### 4.2. Regularization Analysis

We have conducted experiments on the following popular regularization methods: Dropout [50], SpatialDropout [50], and Dropblock [24], as well as our CAR module. We train the network multiple times but change only the regularization method. We set the dropout rate = 0.5, and add regularization into the flow decoder of the network. The results are summarized in Table 1.

We observe that Dropout and Dropblock with a small block size hurt the performance of the network. We think the main reason for this is that the input of the network is a pair of images and the random dropout of pixels misleads the network to mismatch dropout pixels between the images (i.e., pixels with their features set to 0). SpatialDropout improves the error. Different from these methods, our method learns the adaptive content mask for both images and improves the error further.

### 4.3. Comparison to the State-of-the-Art

**Figure 7:**
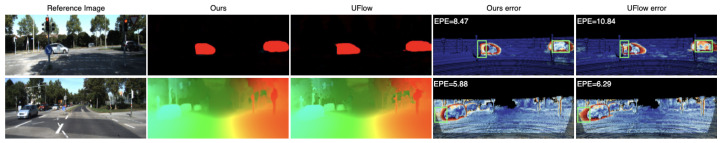
Qualitative visual comparison of our method with UFlow [43] in KITTI 2015. (We did not find the results of UPFlow on the KITTI website.) Red pixels indicate higher errors while blue pixels indicate less errors. Our result is visually improved over the previous state-of-the-art. The green rectangles highlight some obvious areas.

We first compare our method with PWC-Net variants reported on the MPI-Sintel and KITTI benchmarks. Table 2 shows that our method improves on the performance, and that it has lower errors than all other unsupervised PWC-Net methods. On MPI-Sintel, we achieve an AEPE = 4.95 on the final pass which is a 6.9% improvement, and an AEPE = 3.46 on the clean pass which is a 11.3% improvement. We achieve an AEPE = 1.2, which is a 14.2% improvement on KITTI 2012, and Fl-all = 8.40%, which corresponds to a 5.7% improvement on KITTI 2015. We also report supervised methods based on PWC-Net for comparison. Our method with 3 M parameters surpasses classic supervised methods, e.g., PWC-Net with 8.7 M parameters and LiteFlowNet with 5.37 M parameters on the benchmarks. Especially, our method is also comparable with some novel supervised methods such as IRR-PWC [30]. Our method even surpasses some of these supervised methods on KITTI 2012 and on the Sintel Clean pass. Figure 7 shows some qualitative comparisons between our method and previous state-of-the-art methods (For more comparisons, please see Appendix A.3). Our method reduces errors in visual comparison to earlier methods.

We analyze the reasons for the performance improvements, using our method over the comparators. First, our content-aware regularization module helps to break the co-adaptation between channels and learns more discriminative features for optical flow and scene flow estimation. Second, our shared-weight teacher–student strategy enables the simultaneous improvement of both teacher and student models by sharing weights and transferring knowledge. Third, our method benefits from a simple and efficient design that does not require any extra parameters or computation during inference. Our method has fewer parameters, less computation, and a faster inference time than the original PWC-Net. However, as we have chosen to integrate our method into PWC-Lite, we also inherit possible disadvantages, including failures to handle large motions well or errors close to motion boundaries. In future work, we would like to see our method integrated in more powerful optical flow networks, e.g., RAFT [35], but we also note that this can be expected to lead to an increase in model size and computation time.

As can be seen from Table 3, we achieve better a performance than UPflow approximately 8× faster, with about 4× less computation, because we apply our method to a lightweight PWC-Net backbone. Our multi-frame version further improves the accuracy in all benchmarks with less cost than UPFlow. Although the errors of our method are higher than the ones of SMURF, our method leads to a much smaller model and far less computation, and remarkably, is real-time on high-resolution images with competitive accuracy. As discussed in Section 2.2, SMURF also has the serious drawback of being extremely time- and memory-expensive in training, and hence, it is difficult to adapt to new datasets.

### 4.4. Ablation Study

To evaluate the capability of each component of our pipeline, we conducted experiments on the split training datasets of Sintel and KITTI with a small training schedule (300 k iteration with batch size 4). The endpoint error (EPE) of overall pixels (ALL), non-occluded pixels (NOC), and occluded pixels (OCC) are reported for evaluation.

Ablation of the main unsupervised components are reported in Table 4. The main components are: Deformable modulation cost volume (DMCV), level dropout regularization (LDR), and content-aware regularization (CAR). We also include augmentation regularization loss (ARL) [27] to clarify any possible interactions between the modules in our framework.

We start by only using the photometric loss and the smoothness loss to train the network (see Equation (19)). Comparing the first and second row, we can see a significant improvement with our CAR module. Then, we observe that DMCV, LDR, and ARL all improve the network compared to the first row. Comparing all the combinations, we find that all components improve the performance; the combination of ARL and CAR reduces all errors, the accuracy in the occluded region can be improved by LDR, and the multi-frame version can further improve the accuracy. We also analyzed the regularization rate of CAR and LDR, and found that a CAR rate = 0.5 and LDR = 0.9 achieves the highest accuracy (see the Appendix A.1 for details); we have kept this setting for all other training settings.

### 4.5. Cross-Dataset Generalization

To test the generalization ability of a model, we train it only on Sintel raw and final, but evaluate it on the noisy real-world KITTI dataset. Table 5 reports the results of the fully supervised PWC-Net, the unsupervised method ARFlow, and our method. PWC-Net outperforms our method and ARFlow in Sintel which the model is trained on, but this performance does not generalize well to KITTI. Because of the CAR module, the model trained with our unsupervised method generalizes much better. Note that neither of these models have seen real-world images during training.

### 4.6. CAR in Unsupervised Scene Flow Estimation

We extend our method to unsupervised scene flow estimation and implement our CAR module and shared-weight teacher–student strategy in Self-Mono-SF. Self-Mono-SF [68] is an unsupervised Scene Flow method which uses PWC-Net as a backbone. Table 6 reports the results of state-of-the-art unsupervised monocular scene flow methods on the KITTI dataset. We follow the evaluation metric of the KITTI Scene Flow benchmark. D1-all and D2-all are the percentages of stereo disparity outliers in the first frame and in the second frame, respectively. F1-all is the percentage of optical flow outliers. SF1-all is the percentage of scene flow outliers. Our method not only improves on the performance, but also surpasses other unsupervised multi-task methods. We conclude that our method helps with performance in the scene flow estimation task. We suspect that our method is also likely to improve the accuracy in other related dense pixel matching tasks, but leaves further investigations as future work.

## 5. Conclusions

In this paper, we have proposed a novel and effective teacher–student unsupervised learning method for optical flow and scene flow networks. We introduced a content-aware regularization module that randomly enhances and diminishes partial features during training in the teacher model. We showed that our method significantly improves on the performance and generalization of the original networks, and outperforms other popular regularization methods. We also demonstrated that our method can be easily extended to scene flow estimation by adding a depth estimation branch and applying our regularization module to both branches. Our method achieves state-of-the-art results on optical flow and scene flow benchmarks, and shows superior cross-dataset generalization compared to supervised and unsupervised methods. Our method benefits from a simple and efficient design that does not require any extra parameters or computations during inference. Our method has implications for various applications that rely on accurate and robust optical flow and scene flow estimation, such as video analysis, 3D reconstruction, autonomous driving, and robotics.

## Figures and Tables

**Table 1:** End Point Errors (EPEs) of different regularization methods. bs indicates the block size for Dropblock.

Experiment	Sintel Clean	Sintel Final
None	2.79	3.72
Dropout	2.83	3.77
DropBlock, bs = 1	2.81	3.73
DropBlock, bs = 3	2.77	3.75
DropBlock, bs = 7	2.76	3.72
SpatialDropout	2.74	3.69
CAR	2.70	3.63

**Table 2:** MPI Sintel and KITTI official optical flow benchmark results with PWC-Net variants. We report the EPE error (lower is better) to compare the performances for Sintel and KITTI 2012, and the percentage of erroneous pixels F1 (%) error for KITTI 2015. We report on both supervised and unsupervised methods. Missing entries (-) are for values that are not reported for the specific method by the authors. ${}^{‡}$ MDFlow uses RAFT [35] as the student model. The results show that the two frames and the multi-frame versions of our method outperform all unsupervised and even the original supervised PWC-Net, and some other supervised variants.

Method	Sintel Clean		Sintel Final		KITTI-12		KITTI-15	
	Train	Test	Train	Test	Train	Test	Train	Test
PWC-Net [26]	(1.70)	3.86	(2.21)	5.13	(1.45)	1.7	(2.16)	9.60%
IRR-PWC [30]	(1.92)	3.84	(2.51)	4.58	–	–	(1.63)	7.65%
ScopeFlow [56]	-	3.59	-	4.10	–	-	-	6.82%
VCN [34]	(1.66)	2.81	(2.24)	4.40	–	-	(4.1)	6.30%
DICL [33]	(1.11)	2.12	(1.60)	3.44	–	-	(3.6)	6.31%
MaskFlownet [32]	(2.25)	2.52	(3.61)	4.17	(2.94)	1.1	-	6.11%
UnFlow-CSS [67]	–	–	(7.91)	10.22	3.29	–	8.10	23.30%
OccAwareFlow ${}^{†}$ [15]	(4.03)	7.95	(5.95)	9.15	3.55	4.2	8.88	31.2%
UnFlow [67]	–	9.38	(7.91)	10.22	3.29	–	8.10	23.3%
DDFlow [38]	(2.92)	6.18	(3.98)	7.40	2.35	3.0	5.72	14.29%
EpiFlow [41]	(3.54)	7.00	(4.99)	8.51	(2.51)	3.4	(5.55)	16.95%
SelFlow ${}^{†}$ [39]	(2.88)	6.56	(3.87)	6.57	1.69	2.2	4.84	14.19%
ARFlow [27]	(2.79)	4.78	(3.87)	5.89	1.44	1.8	2.85	11.80%
ARFlow-MV ${}^{†}$ [27]	(2.73)	4.49	(3.69)	5.67	1.26	1.5	3.46	11.79%
UFlow [43]	(2.50)	5.21	(3.39)	6.50	1.68	1.9	2.71	11.13%
MDFlow ${}^{‡}$ [44]	(**2.17**)	4.16	(3.14)	5.46	-	-	2.45	8.91%
UPFlow [18]	(2.33)	4.68	(**2.67**)	5.32	1.27	1.4	2.45	9.38%
CAR-Flow (**our**)	(2.36)	3.69	(3.28)	5.21	1.16	1.3	2.34	9.09%
CAR-Flow-MV (**our**) ${}^{†}$	(2.25)	**3.46**	(3.23)	**4.95**	**1.02**	**1.2**	**2.11**	**8.40**%

${}^{†}$ indicates that the method uses multiple (3) frames.

**Table 3:** Evaluation with state-of-the-art methods on MPI Sintel and the KITTI benchmark. Computational cost (FLOPs) and inference time are measured for Sintel images with 448 × 1024 resolution (We tested the inference time on a single Tesla P100 GPU).

Method	# FLOPs	# Params	Inference	Sintel		KITTI	
			Time	Clean	Final	2012	2015
UPFlow [18]	198.27 G	3.49 M	271 ms	4.68	5.32	1.4	9.38%
SMURF ${}^{†}$ [46]	810.14 G	5.26 M	413 ms	3.15	4.18	-	6.38%
CAR-Flow	50.49 G	2.74 M	34 ms	3.69	5.21	1.3	9.09%
CAR-Flow-MV ${}^{†}$	108.08 G	2.97 M	59 ms	3.46	4.95	1.2	8.40%

${}^{†}$ indicates that the method uses multiple (3) frames.

**Table 4:** Ablation study of the main components of our method. EPEs of indicated pixels are reported. **DMCV**: deformable modulation cost volume. **LDR**: level dropout regularization. **ARL**: augmentation regularization loss. **CAR**: content-aware regularization.

DMCV	LDR	ARL	CAR	Sintel Clean			Sintel Final		
				ALL	NOC	OCC	ALL	NOC	OCC
				2.92	1.53	22.14	3.87	2.46	26.24
			✔	2.74	1.32	20.92	3.75	2.31	23.90
✔			✔	2.58	1.17	18.64	3.43	2.14	22.52
		✔	✔	2.53	1.15	18.46	3.51	2.03	22.43
	✔	✔		2.57	1.21	18.72	3.47	1.91	22.16
	✔	✔	✔	2.36	1.04	18.14	3.24	1.77	21.22
✔		✔	✔	2.42	1.16	17.54	3.31	1.84	20.83
✔	✔	✔		2.33	1.11	17.31	3.26	1.81	21.36
✔	✔	✔	✔	2.25	1.01	16.27	3.07	1.62	20.12
✔	✔	✔	✔ ${}^{†}$	**2.13**	**0.99**	**16.12**	**2.83**	**1.55**	**19.70**

${}^{†}$ indicates that the method uses multiple (3) frames.

**Table 5:** Cross-dataset generalization. All methods are trained on Sintel, and they are evaluated on KITTI 2012/2015 training data.

Method	Sintel Clean	Sintel Final	KITTI 2012	KITTI 2015
PWC-Net	(1.86)	(2.31)	3.68	10.52%
ARFlow	(2.79)	(3.73)	3.06	9.04%
CAR-Flow	(2.81)	(3.73)	2.65	7.06%
CAR-Flow-MV ${}^{†}$	(2.22)	(3.26)	2.23	5.97%

${}^{†}$ indicates that the method uses multiple (3) frames.

**Table 6:** Evaluation on KITTI Scene Flow training. Our regularization method clearly reduces the percentage of stereo disparity (D1-all and D2-all), optical flow (F1-all), and sceneflow (SF1-all) outliers compared to its baseline, Self-Mono [68]. Other state-of-the-art multi-task approaches are listed for comparison.

Method	D1-all	D2-all	F1-all	SF1-all
GeoNet [20]	49.54	58.17	37.83	71.32
EPC [69]	26.81	60.97	25.74	(>60.97)
EPC++ [70]	**23.84**	60.32	**19.64**	(>60.32)
Self-Mono-SF [68]	31.25	34.86	23.49	47.0
Self-Mono-SF-CAR (our)	29.24	**32.49**	21.34	**43.57**

**Figure A1:**
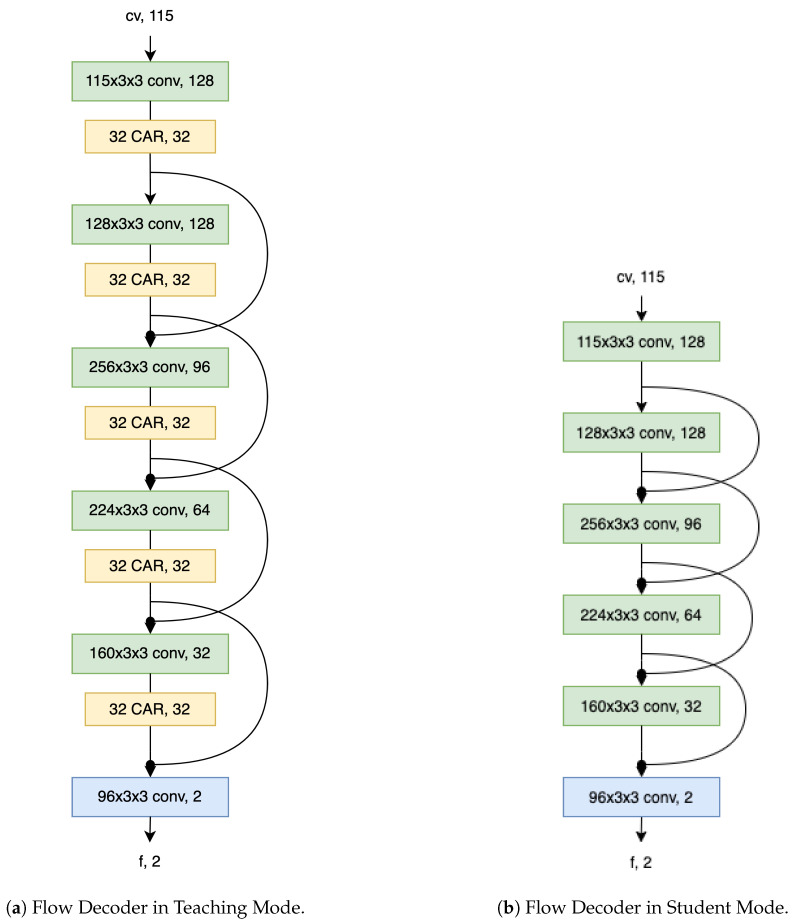
The optical flow decoder network. (**a**) shows flow decoder network in teaching mode with the CAR module active during training. (**b**) shows optical flow decoder network in student mode and during testing (without the CAR module). Except yellow and blue layers, green convolutional layers are followed by a leaky ReLU activation function.

**Figure A2:**
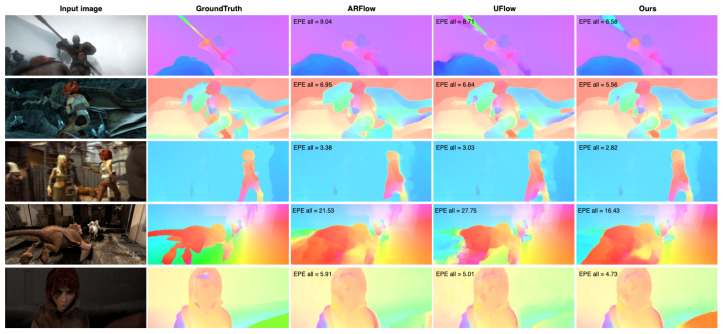
Comparison of flow results on images of the Sintel benchmark. Our method leads to noticably lower EPEs than ARFlow [27] and UFlow [43]. This is also visually noticable at flow boundaries.

**Figure A3:**
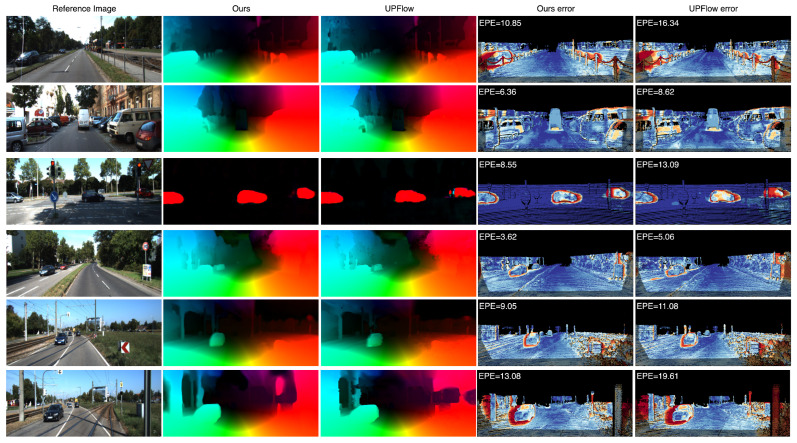
Comparison of flow results on a KITTI-2015 test scene with previous state-of-the-art UPFlow [18]. Our results show a reduced number of outliers, and hence, smoother results, which lead to a large reduction in EPE.

## Data Availability

Only data from public benchmarks were used in this study. See text for the respective references.

## Appendix A

In Appendix A.1, we report an ablation study regarding the probability of invoking the CAR module and LDR during training. In Appendix A.2, we provide the details of the CAR module structure used during the ablation study of CAR. We also present additional examples of the flow results of our method on KITTI-2015 [3] and Sintel [63] in Appendix A.3.

### Appendix A.1. Comparison of the Regularization Rate

We analyze the regularization rates of CAR and LDR. Table A1 shows the probability for invoking CAR or LDR, i.e., a CAR rate = 0.1 means that in each training step, we have a 10% chance to use CAR. Comparing all of the results, we observe that CAR and LDR always help the performance. A CAR rate = 0.5 and LDR = 0.9 achieve the highest accuracy, and we keep this setting for all other training.

**Table A1:** Different rates of CAR and LDR. Rate is the probability that CAR and LDR are invoked in each training step.

	Rate	Sintel Clean	Sintel Final	KITTI-12	KITTI-15
CAR	0.2	2.31	3.24	1.24	2.42
	0.5	**2.23**	3.09	**1**.17	**2.24**
	0.7	2.25	**3.04**	**1.16**	2.28
	0.9	2.29	3.13	1.21	2.37
LDR	0.2	2.33	3.17	1.26	2.32
	0.5	2.21	3.07	1.17	2.21
	0.8	2.19	3.01	1.13	2.18
	0.9	**2.19**	**2.98**	**1.12**	**2.16**

### Appendix A.2. CAR Module Structure

Figure A1a,b shows the detailed implementation of the optical flow decoder network with our CAR module (a two-frame network). In the figures, each layer shows first the numbers of input channels, and last, the numbers of output channels. Our CAR module randomly works on a subset of features; thus, we can use a smaller channel number and the other channels of the features are not affected. The kernel size for the convolutional layers is also given. We uniformly use 3×3 kernels.

### Appendix A.3. Additional Results

Figure A2 shows a visualization of the results of our CAR in comparison with two further methods on Sintel and the state-of-the-art UPFlow [18] on KITTI 2015. Please note that UPFlow on Sintel is compared with our method in the main paper. We can clearly see that previous methods predict flow with noise around object boundaries while the predictions of our method have less outliers, and hence, the flow field is smoother.
